# Supplementary material for: Programmed conversion of hypertrophic chondrocytes into osteoblasts and marrow adipocytes within zebrafish bones
Source: eLife. 2019 Feb 20;8:e42736. doi: 10.7554/eLife.42736 (PMC6398980; doi:10.7554/eLife.42736)
Supplement: Figure 7—source data 1. [file elife-42736-fig7-data1.docx]

| Quantification of Ratio Hypertrophic zone / Proliferative zone (um) | |  |  |  |
| --- | --- | --- | --- | --- |
| 17 mm - WT | 17 mm - *mmp9-/-* | 21 mm - WT | 21 mm - *mmp9-/-* |  |
| 4.491156741 | 3.770848334 | 2.791075608 | 6.508648679 |  |
| 4.314732105 | 2.837304971 | 2.443390009 | 5.949363048 |  |
| 4.491156741 | 3.933736838 | 1.915199294 | 2.694226285 |  |
|  |  | 2.040035626 | 3.213798324 |  |
|  |  |  | 4.752095002 |  |
|  |  |  |  |  |
| Analysis - Prism 7 |  |  |  |  |
| Column B | 17 mm - *mmp9-/-* |  | Column D | 21 mm - *mmp9-/-* |
| vs. | vs. |  | vs. | vs. |
| Column A | 17 mm - WT |  | Column C | 21 mm - WT |
|  |  |  |  |  |
| Unpaired t test |  |  | Unpaired t test |  |
| P value | 0.057 |  | P value | 0.0305 |
| P value summary | ns |  | P value summary | * |
| Significantly different (P < 0.05)? | No |  | Significantly different (P < 0.05)? | Yes |
| One- or two-tailed P value? | Two-tailed |  | One- or two-tailed P value? | Two-tailed |
| t, df | t=2.65 df=4 |  | t, df | t=2.704 df=7 |
|  |  |  |  |  |
| How big is the difference? |  |  | How big is the difference? |  |
| Mean ± SEM of column A | 4.432 ± 0.05881, n=3 |  | Mean ± SEM of column C | 2.297 ± 0.1994, n=4 |
| Mean ± SEM of column B | 3.514 ± 0.3416, n=3 |  | Mean ± SEM of column D | 4.624 ± 0.7429, n=5 |
| Difference between means | -0.9184 ± 0.3466 |  | Difference between means | 2.326 ± 0.8604 |
| 95% confidence interval | -1.881 to 0.04395 |  | 95% confidence interval | 0.2917 to 4.361 |
| R squared (eta squared) | 0.637 |  | R squared (eta squared) | 0.5108 |
|  |  |  |  |  |
| F test to compare variances |  |  | F test to compare variances |  |
| F, DFn, Dfd | 33.74, 2, 2 |  | F, DFn, Dfd | 17.34, 4, 3 |
| P value | 0.0576 |  | P value | 0.0412 |
| P value summary | ns |  | P value summary | * |
| Significantly different (P < 0.05)? | No |  | Significantly different (P < 0.05)? | Yes |

| Quantification of Growth Plate Width (um) |  |  |  |  |  |  |  |  |
| --- | --- | --- | --- | --- | --- | --- | --- | --- |
| WT | *mmp9-/-* | WT->*mmp9-/-:* +BFP clone | WT->mmp9-/-: -BFP clone | WT->WT |  |  |  |  |
| 183.5088355 | 409.0413152 | 120.2153006 | 151.0298589 | 114.2811122 |  |  |  |  |
| 192.970517 | 441.6939819 | 129.5360434 | 187.849746 | 131.4702586 |  |  |  |  |
| 206.2549916 | 315.7500285 | 247.331394 | 141.214478 | 169.1304085 |  |  |  |  |
| 170.0686573 | 380.4845605 | 178.907425 | 357.213251 | 143.0331275 |  |  |  |  |
| 161.4016013 | 346.1617707 | 291.9991293 | 327.1748595 | 165.3892583 |  |  |  |  |
| 157.2150522 | 352.2275354 | 124.7892425 | 193.0539064 | 103.7698884 |  |  |  |  |
| 171.2699915 |  | 217.2108763 | 428.5591308 | 130.1185387 |  |  |  |  |
| 142.9802485 |  | 134.1971424 | 340.4130056 | 184.4761735 |  |  |  |  |
|  |  |  |  | 154.7791498 |  |  |  |  |
|  |  |  |  | 160.5804243 |  |  |  |  |
|  |  |  |  |  |  |  |  |  |
|  |  |  |  |  |  |  |  |  |
| Analysis - Prism 7 |  |  |  |  |  |  |  |  |
| Number of families | 1 |  |  |  |  |  |  |  |
| Number of comparisons per family | 10 |  |  |  |  |  |  |  |
| Alpha | 0.05 |  |  |  |  |  |  |  |
|  |  |  |  |  |  |  |  |  |
| Tukey's multiple comparisons test | Mean Diff. | 95.00% CI of diff. | Significant? | Summary | Adjusted P Value | |  |  |
|  |  |  |  |  |  |  |  |  |
| WT vs. mmp9-/- | -201 | -296.8 to -105.2 | Yes | **** | <0.0001 | A-B |  |  |
| WT vs. WT->mmp9-/-: +BFP clone | -7.315 | -96.04 to 81.41 | No | ns | 0.9993 | A-C |  |  |
| WT vs. WT->mmp9-/-: -BFP clone | -92.6 | -181.3 to -3.884 | Yes | * | 0.0372 | A-D |  |  |
| WT vs. WT->WT | 27.51 | -56.66 to 111.7 | No | ns | 0.8794 | A-F |  |  |
| mmp9-/- vs. WT->mmp9-/-: +BFP cloneP | 193.7 | 97.87 to 289.5 | Yes | **** | <0.0001 | B-C |  |  |
| mmp9-/- vs. WT->mmp9-/-: -BFP clone | 108.4 | 12.58 to 204.2 | Yes | * | 0.02 | B-D |  |  |
| mmp9-/- vs. WT->WT | 228.5 | 136.9 to 320.2 | Yes | **** | <0.0001 | B-F |  |  |
| WT->mmp9-/-: +BFP clone vs. WT->mmp9-/-: -BFP clone | -85.29 | -174 to 3.431 | No | ns | 0.0644 | C-D |  |  |
| WT->mmp9-/-: +BFP clone vs. WT->WT | 34.82 | -49.35 to 119 | No | ns | 0.7573 | C-F |  |  |
| WT->mmp9-/-: -BFP clone vs. WT->WT | 120.1 | 35.94 to 204.3 | Yes | ** | 0.002 | D-F |  |  |
|  |  |  |  |  |  |  |  |  |
|  |  |  |  |  |  |  |  |  |
| Test details | Mean 1 | Mean 2 | Mean Diff. | SE of diff. | n1 | n2 | q | DF |
|  |  |  |  |  |  |  |  |  |
| WT vs. mmp9-/- | 173.2 | 374.2 | -201 | 33.33 | 8 | 6 | 8.529 | 35 |
| WT vs. WT->mmp9-/-: +BFP clone | 173.2 | 180.5 | -7.315 | 30.86 | 8 | 8 | 0.3352 | 35 |
| WT vs. WT->mmp9-/-: -BFP clone | 173.2 | 265.8 | -92.6 | 30.86 | 8 | 8 | 4.244 | 35 |
| WT vs. WT->WT | 173.2 | 145.7 | 27.51 | 29.28 | 8 | 10 | 1.329 | 35 |
| mmp9-/- vs. WT->mmp9-/-: +BFP clone | 374.2 | 180.5 | 193.7 | 33.33 | 6 | 8 | 8.219 | 35 |
| mmp9-/- vs. WT->mmp9-/-: -BFP clone | 374.2 | 265.8 | 108.4 | 33.33 | 6 | 8 | 4.6 | 35 |
| mmp9-/-vs. WT->WT | 374.2 | 145.7 | 228.5 | 31.87 | 6 | 10 | 10.14 | 35 |
| WT->mmp9-/-: +BFP clone vs. WT->mmp9-/-: -BFP clone | 180.5 | 265.8 | -85.29 | 30.86 | 8 | 8 | 3.909 | 35 |
| WT->mmp9-/-: +BFP clone vs. WT->WT | 180.5 | 145.7 | 34.82 | 29.28 | 8 | 10 | 1.682 | 35 |
| WT->mmp9-/-: -BFP clone vs. WT->WT | 265.8 | 145.7 | 120.1 | 29.28 | 8 | 10 | 5.802 | 35 |

| Quantification of Adipocyte # / Marrow Area per 100 um | | |  |  |  |  |  |  |
| --- | --- | --- | --- | --- | --- | --- | --- | --- |
| WT | *mmp9-/-* | WT->*mmp9-/-* | WT->WT |  |  |  |  |  |
| 2.65123 | 1.18708 | 1.21349 | 1.38954 |  |  |  |  |  |
| 2.53249 | 1.01946 | 1.77575 | 1.81471 |  |  |  |  |  |
| 1.60483 | 1.59006 | 2.243284649 | 2.328183716 |  |  |  |  |  |
| 1.75194 | 1.45779 | 1.717719011 | 2.176320047 |  |  |  |  |  |
| 1.80447 | 1.16424 | 2.505976096 | 2.383398324 |  |  |  |  |  |
| 1.61786 | 1.1471 | 1.244261413 | 2.115981092 |  |  |  |  |  |
|  |  | 2.431380797 | 2.003878247 |  |  |  |  |  |
|  |  |  |  |  |  |  |  |  |
|  |  |  |  |  |  |  |  |  |
| Analysis - Prism 7 |  |  |  |  |  |  |  |  |
| Number of families | 1 |  |  |  |  |  |  |  |
| Number of comparisons per family | 6 |  |  |  |  |  |  |  |
| Alpha | 0.05 |  |  |  |  |  |  |  |
|  |  |  |  |  |  |  |  |  |
| Tukey's multiple comparisons test | Mean Diff. | 95.00% CI of diff. | Significant? | Summary | Adjusted P Value | |  |  |
|  |  |  |  |  |  |  |  |  |
| WT vs. mmp9-/- | 0.7328 | 0.07045 to 1.395 | Yes | * | 0.0265 | A-B |  |  |
| WT vs. WT->mmp9-/- | 0.1178 | -0.5205 to 0.7561 | No | ns | 0.9552 | A-C |  |  |
| WT vs. WT->WT | -0.03648 | -0.6748 to 0.6018 | No | ns | 0.9985 | A-D |  |  |
| mmp9-/- vs. WT->mmp9-/- | -0.615 | -1.253 to 0.02328 | No | ns | 0.0617 | B-C |  |  |
| mmp9-/- vs. WT->WT | -0.7693 | -1.408 to -0.131 | Yes | * | 0.0144 | B-D |  |  |
| WT->mmp9-/- vs. WT->WT | -0.1543 | -0.7676 to 0.459 | No | ns | 0.8965 | C-D |  |  |
|  |  |  |  |  |  |  |  |  |
|  |  |  |  |  |  |  |  |  |
| Test details | Mean 1 | Mean 2 | Mean Diff. | SE of diff. | n1 | n2 | q | DF |
|  |  |  |  |  |  |  |  |  |
| WT vs. mmp9-/- | 1.994 | 1.261 | 0.7328 | 0.2385 | 6 | 6 | 4.345 | 22 |
| WT vs. WT->mmp9-/- | 1.994 | 1.876 | 0.1178 | 0.2299 | 6 | 7 | 0.7249 | 22 |
| WT vs. WT->WT | 1.994 | 2.03 | -0.03648 | 0.2299 | 6 | 7 | 0.2245 | 22 |
| mmp9-/- vs. WT->mmp9-/- | 1.261 | 1.876 | -0.615 | 0.2299 | 6 | 7 | 3.784 | 22 |
| mmp9-/-vs. WT->WT | 1.261 | 2.03 | -0.7693 | 0.2299 | 6 | 7 | 4.733 | 22 |
| WT->mmp9-/- vs. WT->WT | 1.876 | 2.03 | -0.1543 | 0.2209 | 7 | 7 | 0.9881 | 22 |
